# Oral health conditions and cognitive functioning in middle and later adulthood

**DOI:** 10.1186/1472-6831-14-70

**Published:** 2014-06-13

**Authors:** Stefan Listl

**Affiliations:** 1 Department of Conservative Dentistry, University of Heidelberg, Heidelberg, Germany; 2 Max-Planck-Institute for Social Law and Social Policy, Munich Center for the Economics of Aging, Munich, Germany

**Keywords:** Cognitive functioning, Chewing ability, Aging

## Abstract

**Background:**

The purpose of the present study was to examine the impact of oral health conditions on cognitive functioning on basis of data samples from several European countries.

**Methods:**

Secondary analyses were conducted of data from wave 2 of the Survey of Health, Ageing, and Retirement in Europe (SHARE) which includes 14 European countries and is intended to be representative of each country’s middle and later adulthood population. Information on word recall, verbal fluency, and numeracy as well as information on chewing ability and denture wearing status was available for a total of 28,693 persons aged 50+. Multivariate regression analysis was used to detect influences of oral health parameters on cognitive functioning (p < 0.05).

**Results:**

Persons with good chewing ability or without dentures had significantly better word recall, verbal fluency, and numeracy skills than persons with chewing impairment or with dentures. The observed patterns of parameter estimates imply differential oral health impacts on numeracy compared to word recall and verbal fluency.

**Conclusions:**

The present study provides novel large-scale epidemiological evidence supportive of an association between oral health and cognitive functioning. Future research should intend to verify the precise causal links between oral health conditions, various cognitive dimensions, and their neural correlates.

## Background

There is a growing literature which suggests that cognitive functioning is influenced by oral health status [1-7]. Understanding the nature and magnitude of such dependencies is particularly relevant in times of population ageing because there is conclusive evidence about cognitive functions to decline with older age [8-10]. Cognitive performance has been shown to be pivotal for persons’ daily activities and its deterioration is considered an important predictor of dementia and a close correlate of worsening health in general [8,11-14]. Maintenance of good oral health may thus provide a relevant intervention point to foster vivid cognitive functioning and successful aging up until older adulthood.

Experimental evidence indicates that tooth loss and limited chewing ability are associated with compromised learning and memory capacities [1]. Potential pathways have been proposed accordingly. In response to mastication, increased cerebral blood flows and higher oxygen levels were observed within the central nervous system, notably the hippocampus and the prefrontal cortex [15-17]. Moreover, it is suggested that neuronal signals from teeth influence hippocampus functions [18,19]. Within the central nervous system, the hippocampus and the prefrontal cortex are considered important for learning and memory; the prefrontal cortex is also viewed as being relevant for numeracy [20,21].

Yet the epidemiological evidence about the relationship between oral health and cognitive functioning remains limited and few studies have been based on country-representative data. Notable exceptions include a study focusing on the oldest old in Sweden [7] and a US-NHANES study on periodontal disease [22]. Specifically, there is a dearth of epidemiological evidence with comprehensive inclusion of middle and later adulthood populations from various countries. Such information may be important to better conceive the overall extent to which oral health conditions influence cognitive functioning. Moreover, previous studies have mainly been looking at aggregate measures of cognitive impairment or examined only learning and memory skills. In contrast, although day-to-day decision making relies extensively on abilities to process numbers and calculations [23,24], it is still unclear whether numeracy might also be influenced by oral health status.

Therefore, the purpose of the present study was to examine, on basis of unique large-scale survey data representative of several middle and later adulthood populations in Europe, the extent to which learning, memory, and numeracy skills at age 50+ are influenced by oral health conditions. It was hypothesized that good chewing ability and not wearing dentures are associated with favorable word recall, verbal fluency, and numeracy skills.

## Methods

The present paper is based on data from wave 2 of the Survey of Health, Ageing, and Retirement in Europe (SHARE) which were collected in 2006–2007. SHARE contains extensive information on health, social environments, and living conditions of persons aged 50 years and older from 14 European countries (Austria, Belgium, Czech Republic, Denmark, France, Germany, Greece, Ireland, Italy, Netherlands, Poland, Spain, Sweden, and Switzerland). The SHARE survey generally relies on self-completed paper-pencil questionnaires and computer-assisted personal interviews. SHARE samples were drawn to be representative of each examined country’s middle and later adulthood population. Further details about the SHARE methodology are available via http://www.share-project.org and are described in the literature [25].

This study employs SHARE wave 2 information on various aspects of respondents’ cognitive functioning, that is word recall, verbal fluency, and numeracy. For testing persons’ word recall skills, respondents were introduced to the respective task as follows: “*Now, I am going to read a list of words from my computer screen. We have purposely made the list long so it will be difficult for anyone to recall all the words. Most people recall just a few. Please listen carefully, as the set of words cannot be repeated. When I have finished, I will ask you to recall aloud as many of the words as you can, in any order. Is this clear?*” Then, a list consisting of ten words (*butter, arm, letter, queen, ticket, grass, corner, stone, book, stick*) was provided to respondents. For testing persons’ immediate word recall, up to one minute recall time was allowed after provision of the word list to name as many words as respondents could remember. For testing persons’ delayed word recall, respondents were again asked to name as many as possible out of the ten words list after completion of five other SHARE questions. Based on the information about how many out of the ten words could be recalled, two corresponding variables were constructed for immediate word recall (variable “Word Recall A”) and delayed word recall (variable “Word Recall B”).

For testing persons’ verbal fluency, the following instruction was given to respondents: “*Now I would like you to name as many different animals as you can think of. You have one minute to do this. Ready, go*”. Accordingly, a variable was constructed which contains the number of different animals named by the respondent (variable “Verbal Fluency”).

For testing persons’ numeracy, the present study relies on a test score based on the following questions which were directed to SHARE respondents:

– “*If the chance of getting a disease is 10 per cent, how many people out of 1000 (one thousand) would be expected to get the disease?*” [question 1]

– *“In a sale, a shop is selling all items at half price. Before the sale, a sofa costs 300 [{local currency}]. How much will it cost in the sale?”* [question 2]

– *“A second hand car dealer is selling a car for 6,000 [{local currency}]. This is two-thirds of what it costs new. How much did the car cost new?”* [question 3]

– “*Let’s say you have 2000 [{local currency}] in a savings account. The account earns ten per cent interest each year. How much would you have in the account at the end of two years?*” [question 4]

The corresponding numeracy test score (variable “Numeracy”) ranges from 0 to 4 and is constructed as follows: all respondents are asked question 1. If question 1 is answered incorrectly, question 2 is asked and the test ends. If question 2 is answered incorrectly, the numeracy test score is 0. Answering question 2 correctly gives a test score of 1. Respondents who answered question 1 correctly continue with question 3. If this is answered incorrectly, the final test score is 2. If question 3 is answered correctly, question 4 is asked additionally. If question 4 is answered incorrectly, the test score is 3. Finally, if question 4 is answered correctly, the test score is 4.

Each of the described variables for cognitive functioning (Word Recall A, Word Recall B, Verbal Fluency, Numeracy) served as a dependent variable in multivariate Ordinary Least Square (OLS) regression analysis. In order to detect dependencies between oral health and cognitive functioning and to control for socio-demographic and general health influences, the items described hereafter were used as explanatory variables.

Oral health measures in SHARE include binary variables for chewing ability (whether or not respondents are able to bite/chew on hard foods) and denture wearing (whether or not respondents wear dentures). Chewing ability and denture wearing are often considered relevant correlates of oral health and the according SHARE variables have been used within a number of previous studies as measures of subjective oral health status or as proxy for tooth loss, respectively [26-33]. Besides age, sex, and country of residence, respondents’ educational attainment and job situation were used as socio-demographic controls. In SHARE, educational attainment is classified according to the UNESCO International Standard Classification of Education (ISCED) [34]. The according control variable distinguishes between (pre-)primary (ISCED scores 0 and 1), secondary (ISCED scores 2 and 3), and postsecondary education (ISCED scores 4–6). The control variable for persons’ job situation indicates whether respondents are retired, employed or self-employed, unemployed and looking for work, permanently sick or disabled, or homemakers. In terms of general health, SHARE includes a self-rated measure distinguishing between poor, fair, good, very good, and excellent health. Further control variables for health are grip strength as measured on the dominant hand (in kilograms) and a binary measure indicating whether or not a person had felt sad or depressed within the past month. Data analysis was carried out in STATA/SE version 12.0 (StataCorp, College Station, TX). Adjustments were neither made for unit non-response (complete absence of interview) nor for item non-response (absence of answers to specific questions in the interview after agreement to participate in the survey).

## Results

Table 1 shows summary statistics of dependent variables measuring cognitive functioning by respondents’ country of residence. In response to the immediate word recall task (Word Recall A), the average number of words given correctly ranged from 5.6 [SD:1.8] in Denmark to 3.7 [1.8] in Spain. In response to the delayed word recall task (Word Recall B), the average number of words given correctly ranged from 4.3 [2.0] in Denmark to 2.5 [1.7] in Spain. For the verbal fluency task, the average number of different animals named within one minute ranged from 23.1 [SD:7.6] in Sweden to 14.3 [6.8] in Spain. Finally, in response to the numeracy task, the average test score ranged from 3.0 [0.7] in Switzerland to 2.2 [0.5] in Spain.

**Table 1:** Summary statistics of dependent variables measuring cognitive functioning

	**Word recall A**	**Word recall B**	**Verbal fluency**	**Numeracy**	
	***Immediate recall***	***Delayed recall***	***Animal names***	***Test score***	
	**Mean (SD) of words**	**Mean (SD) of words**	**Mean (SD) of names**	**Mean (SD) of score**	
	**[range: 0–10]**	**[range: 0–10]**	**[range: 0–100]**	**[range: 0–4]**	
Austria	5.4 (2.0)	4.0 (2.2)	21.0 (8.2)	2.9 (0.7)	
	[n = 1,313]	[n = 1,313]	[n = 1,304]	[n = 1,315]	
Germany	5.4 (1.7)	3.8 (1.9)	21.3 (7.5)	2.9 (0.8)	
	[n = 2,466]	[n = 2,466]	[n = 2,457]	[n = 2,492]	
Sweden	5.4 (1.7)	4.2 (2.0)	23.1 (7.6)	2.8 (0.8)	
	[n = 2,623]	[n = 2,624]	[n = 2,622]	[n = 2,655]	
Netherlands	5.3 (1.7)	4.0 (2.1)	20.1 (6.5)	2.9 (0.8)	
	[n = 2,556]	[n = 2,556]	[n = 2,542]	[n = 2,584]	
Spain	3.7 (1.8)	2.5 (1.7)	14.3 (6.8)	2.2 (0.5)	
	[n = 2,117]	[n = 2,118]	[n = 2,082]	[n = 2,145]	
Italy	4.4 (1.9)	3.0 (2.1)	15.1 (7.8)	2.4 (0.6)	
	[n = 2,869]	[n = 2,869]	[n = 2,850]	[n = 2,889]	
France	4.7 (1.9)	3.2 (2.0)	19.7 (7.9)	2.5 (0.7)	
	[n = 2,723]	[n = 2,727]	[n = 2,683]	[n = 2,786]	
Denmark	5.6 (1.8)	4.3 (2.0)	22.0 (7.2)	2.8 (0.8)	
	[n = 2,477]	[n = 2,478]	[n = 2,473]	[n = 2,498]	
Greece	4.8 (1.6)	3.3 (1.7)	14.5 (5.1)	2.7 (0.7)	
	[n = 3,007]	[3,008]	[n = 2,987]	[n = 2,984]	
Switzerland	5.3 (1.7)	3.9 (2.0)	21.1 (6.7)	3.0 (0.7)	
	[n = 1,404]	[n = 1,404]	[n = 1,404]	[n = 1,409]	
Belgium	5.1 (1.8)	3.5 (2.0)	20.2 (6.6)	2.6 (0.7)	
	[n = 2,998]	[n = 2,999]	[n = 2,980]	[n = 2,999]	
Czech Republic	5.1 (1.7)	3.4 (1.9)	19.8 (7.6)	2.7 (0.7)	
	[n = 2,692]	[n = 2,692]	[n = 2,684]	[n = 2,714]	
Poland	4.3 (1.8)	2.8 (1.9)	15.5 (6.6)	2.4 (0.6)	
	[n = 2,377]	[n = 2,377]	[n = 2,376]	[n = 2,357]	
Ireland	5.3 (1.8)	4.1 (2.3)	16.1 (6.4)	2.7 (0.7)	
	[n = 1,104]	[n = 1,104]	[n = 1,101]	[n = 1,132]	

**NB:** data from SHARE wave 2; results are weighted (except for Ireland for which SHARE does not provide a weighting variable).

Table 2 displays summary statistics of the explanatory variables. About 78% of respondents were able to bite or chew on hard foods and about 42% of respondents had a denture. Average age of respondents was 66 years and 55% were women. One third of the sample had an educational attainment corresponding to ISCED scores 0–1, 48% had an ISCED score of 2–3 and about 19% had an ISCED score of 4–6. More than half of respondents were retired, about a quarter of persons were employed or self-employed, 3% unemployed and looking for work, 4% permanently sick or disabled, and 14% were homemakers. 13% of persons reported to have poor general health, 28% to have fair health, 38% to have good health, 14% to have very good health, and 6% to have excellent general health. Average grip strength of respondents was 32.6 kg. About 42% of persons reported to have been sad or depressed in the past month.

**Table 2:** Summary statistics of explanatory variables

	**Mean/proportion**	**Std. dev.**^**#**^	**Min**	**Max**	**N**
Chewing ability					
…can bite/chew on hard foods	77.6%	-	0	1	32,079
…can not bite/chew on hard foods	22.4%	-	0	1	32,079
Wearing dentures					
…yes	41.8%	-	0	1	32,086
…no	58.2%	-	0	1	32,086
Age (years)	66.1	10.5	50	104	32,197
Sex					
…woman	54.8%	-	0	1	32,198
…man	45.2%	-	0	1	32,198
Educational attainment					
…ISCED scores 0-1	32.8%	-	0	1	31,511
…ISCED scores 2-3	48.1%	-	0	1	31,511
…ISCED scores 4-6	19.1%	-	0	1	31,511
Job situation					
…retired	51.7%	-	0	1	31,816
…employed or self-employed	25.5%	-	0	1	31,816
…unemployed and looking for work	3.2%	-	0	1	31,816
…permanently sick or disabled	3.9%	-	0	1	31,816
…homemaker	14.1%	-	0	1	31,816
…other	1.6%	-	0	1	31,816
General health (self-rated)					
…poor	13.1%	-	0	1	32,085
…fair	28.2%	-	0	1	32,085
…good	38.1%	-	0	1	32,085
…very good	14.2%	-	0	1	32,085
…excellent	6.4%	-	0	1	32,085
Grip strength (in kg; dominant hand)	32.6	12.4	0	80	28,969
Sad or depressed					
…yes	41.7%	-	0	1	31,693
…no	58.3%	-	0	1	31,693

**NB:** data from SHARE wave 2; results are weighted (Ireland excluded as SHARE does not provide a weighting variable);

^#^standard deviations are not reported for binary variables.

Table 3 shows results from regression analyses on items of cognitive functioning. The shown parameter estimates originate from multivariate OLS regression models in which all variables listed in the table and additional control variables for age, sex, as well as country dummy variables were simultaneously entered. Parameter estimates in columns 1 and 2 indicate that persons with uncompromised chewing ability remember significantly more words in both the immediate and delayed word recall task than persons who cannot bite or chew on hard foods. Similarly, persons who do not wear dentures perform better in both the immediate and the delayed word recall task than persons with dentures. Parameter estimates in column 3 indicate that verbal fluency is significantly higher amongst persons who can bite or chew on hard foods (relative to persons who cannot) and amongst persons without dentures (compared with denture wearers). Moreover, parameter estimates in column 4 indicate that persons with good chewing ability and persons who do not wear dentures perform better in terms of numeracy. For both word recall and verbal fluency, the magnitude of parameter estimates for good chewing ability is non-significantly greater than the parameter estimates for not wearing dentures. Contrarily, the magnitude of the parameter estimate for absence of denture wearing is greater that of the parameter estimate for good chewing ability, though again without statistical significance.

**Table 3:** Results from multivariate OLS regression analyses on items of cognitive functioning

	***Word recall A***	***Word recall B***	***Verbal fluency***	***Numeracy***
Chewing ability				
…can not bite/chew on hard foods	(reference)	(reference)	(reference)	(reference)
…can bite/chew on hard foods	0.18* [0.13;0.22]	0.14* [0.09;0.20]	0.45* [0.26;0.65]	0.05* [0.03;0.07]
Wearing dentures				
…yes	(reference)	(reference)	(reference)	(reference)
…no	0.13* [0.09;0.17]	0.13* [0.08;0.18]	0.27* [0.10;0.43]	0.08* [0.06;0.09]
Educational attainment				
…ISCED scores 0-1	(reference)	(reference)	(reference)	(reference)
…ISCED scores 2-3	0.60* [0.55;0.64]	0.55* [0.50;0.61]	1.94* [1.76;2.11]	0.27* [0.25;0.29]
…ISCED scores 4-6	0.98* [0.93;1.04]	0.99* [0.93;1.05]	4.07* [3.85;4.30]	0.52* [0.50;0.55]
Job situation				
…retired	(reference)	(reference)	(reference)	(reference)
…employed or self-employed	-0.11* [-0.16;-0.06]	-0.10* [-0.16;-0.04]	0.10 [-0.14;0.33]	0.03* [0.00;0.05]
…unemployed and looking for work	-0.23* [-0.35;-0.11]	-0.26* [-0.40;-0.12]	-0.87* [-1.35;-0.39]	-0.10* [-0.15;-0.05]
…permanently sick or disabled	-0.19* [-0.30; -0.08]	-0.10 [-0.22;0.03]	-0.27 [-0.68;0.14]	-0.06* [-0.11;-0.01]
…homemaker	-0.11* [-0.18;-0.05]	-0.15* [-0.22;-0.07]	-0.32* [-0.57;-0.08]	-0.06* [-0.08;-0.03]
…other	-0.12 [-0.27;0.04]	-0.12 [-0.30;0.05]	-0.38 [-1.18;0.42]	-0.04 [-0.11;0.03]
General health (self-rated)				
…poor	(reference)	(reference)	(reference)	(reference)
…fair	0.23* [0.15;0.30]	0.20* [0.12;0.28]	0.69* [0.40;0.98]	0.03 [-0.00;0.05]
…good	0.39* [0.32;0.47]	0.39* [0.31;0.47]	1.36* [1.07;1.65]	0.08* [0.05;0.10]
…very good	0.50* [0.42;0.58]	0.53* [0.43;0.62]	2.01* [1.68;2.33]	0.19* [0.15;0.22]
…excellent	0.52* [0.43;0.61]	0.52* [0.41;0.62]	2.34* [1.96;2.72]	0.16* [0.12;0.20]
Grip strength	0.01* [0.01;0.02]	0.01* [0.01;0.02]	0.08* [0.07;0.09]	0.01* [0.01;0.01]
Sad or depressed				
…no	(reference)	(reference)	(reference)	(reference)
…yes	-0.07* [-0.11;-0.03]	-0.09* [-0.13;-0.04]	0.27* [0.11;0.43]	-0.04* [-0.06;-0.02]
Number of observations	29,037	29,041	29,020	28,756

**NB:** 95%-Confidence-Intervals in brackets;* indicates statistical significance at least at the 5% level; parameter estimates originate from OLS regression models in which all variables listed above and additional control variables for age, sex, as well as country dummy variables are simultaneously entered.

As for other parameter estimates in Table 3, there is a significant increase in cognitive functioning with higher educational attainment. In comparison with retired respondents, there is a tendency towards cognitive functioning being lower amongst persons who are unemployed and looking for work, those who are permanently sick or disabled, and homemakers; employed or self-employed persons perform significantly worse in the word recall tasks than retired persons but significantly better in the numeracy task. There is also a significant increase in cognitive functioning with better general health and higher grip strength. Persons with recent sadness or depression perform worse than persons without such experiences in terms of word recall and numeracy but better in terms of verbal fluency.

## Discussion

Based on large-scale survey data from SHARE wave 2, the present study provides novel evidence for the association between oral health and cognitive functioning. Findings indicate that persons with good chewing ability or without dentures have significantly better word recall, verbal fluency, and numeracy skills than persons with chewing impairment or with dentures.

The results of this study are generally supportive of findings from previous experimental and smaller scale epidemiological studies. In particular, the examined immediate and delayed word recall tests can be interpreted as verbal learning experiments. Moreover, the researched verbal fluency task represents a semantic memory test. Word recall and verbal fluency were both found to be significantly better in case of good chewing ability and in the absence of denture wearing, the latter resembling a reasonable proxy for tooth loss. Accordingly, the findings of the present study corroborate the concept that reduced learning and memory skills are associated with compromised chewing ability and tooth loss.

A specific novelty of the present study was its explicit examination of numeracy skills. Similarly to the findings for word recall and verbal fluency, numeracy was found to be significantly better in case of good chewing ability and in the absence of denture wearing. Yet, other than for word recall and verbal fluency, the impact of denture wearing on numeracy was somewhat larger than the impact of chewing ability. One interpretation of such a pattern could be that oral health influences on numeracy do not share fully identical mechanisms compared to learning and memory. In order to better understand the precise causal links, future research is therefore encouraged to further investigate the connections between oral health conditions, various cognitive dimensions, and their neural correlates. Closer examination of numeric skills may be particularly worthwhile because such skills are not only considered relevant for decision making in general [23,24] but also specifically for the perception of health risks and the ability to make reasonable medical decisions [35,36].

Some limitations of the present study should be mentioned. First, the results cannot directly be compared with previous studies which have used other measures of cognitive functioning such as the Mini-Mental State Examination (MMSE) [37]. Notwithstanding this, the employed measures comprehensively capture various dimensions of cognitive functioning and are relevant in their own right. For example, the 10-word recall test used in the present study forms routine part of the Telephone Interview for Cognitive Status (TICS-M) and allows higher discriminatory power than the 3-word recall test of the MMSE [38]. Second, the oral health measures used, i.e. chewing ability and denture wearing, may be considered only proxies but were the only oral health information available in SHARE. Note that SHARE was designed to cover a wide variety of persons’ health and living conditions and thus cannot meet the same standards as studies specifically designed to measure oral health. Yet the general relevance of SHARE oral health measures has been documented through earlier research [26-33]. Third, despite the present study identified significant associations between oral health conditions and cognitive outcomes, the respective effect sizes may be considered relatively small. In addition, word recall, verbal fluency, and numeracy were measured on different scales and this makes direct comparisons between the effect sizes of parameter estimates for the association between oral health markers and various cognitive outcome measures difficult. Moreover, the present study relied on survey-based cross-sectional data which may imply some inaccuracy of persons’ responses and doesn’t lend itself to direct causal inference. Unit non-response and item-non-response are frequently considered limitations in survey based research. In this context, it should be noted that no interview could be obtained from about 38% of SHARE households which were initially sampled for interview [25]. Also, SHARE wave 2 was intended to be representative of the middle and later adulthood population in each of the included 14 countries rather than the entire European population. Given the scarcity of other comparable large-scale epidemiological data sources, however, SHARE offers a unique possibility to study the relationship between oral health and various dimensions of cognitive functioning on basis of multi-country data representing several middle and late adulthood populations.

## Conclusions

The present study provides novel large-scale epidemiological evidence supportive of an association between oral health and cognitive functioning. Future research should intend to verify the precise causal links between oral health conditions, various cognitive dimensions, and their neural correlates.

## Competing interests

The author declares that there is no conflict of interests.

## Pre-publication history

The pre-publication history for this paper can be accessed here:

http://www.biomedcentral.com/1472-6831/14/70/prepub
